# Identifying potential pathogenesis and immune infiltration in diabetic foot ulcers using bioinformatics and in vitro analyses

**DOI:** 10.1186/s12920-023-01741-2

**Published:** 2023-12-01

**Authors:** Yuanyuan Xu, Jianchang Xu, Sirong Chen, Anbang Zhou, Guangjing Huang, Shidao Huang, Dianbo Yu, Biaoliang Wu

**Affiliations:** 1 Graduate School, Youjiang Medical University for Nationalities, Baise, 533000 Guangxi China; 2 Department of Endocrinology, The Affiliated Hospital of Youjiang Medical University for Nationalities, Baise, 533000 Guangxi China; 3 The First Clinical College of Wuhan University, Wuhan, 430000 China; 4 Department of Orthopedics, The Affiliated Hospital of Youjiang Medical University for Nationalities, Baise, 533000 Guangxi China

**Keywords:** Diabetic foot ulcers, miRNAs-mRNA, Immune infiltration, Dual-luciferase reporter assay, qPCR, Immunofluorescence staining

## Abstract

**Background:**

Diabetic foot ulcers (DFU) are among the fastest-growing diseases worldwide. Recent evidence has emphasized the critical role of microRNA (miRNA)-mRNA networks in various chronic wounds, including DFU. In this study, we aimed to clarify the miRNA-mRNA axes associated with the occurrence of DFU.

**Methods:**

Expression profiles of miRNAs and mRNAs were extracted from the Gene Expression Omnibus. Differentially expressed genes and differentially expressed miRNAs were identified, and miRNA-mRNA regulatory axes were constructed through integrated bioinformatics analyses. We validated the miRNA-mRNA axes using quantitative real-time PCR (qPCR) and dual-luciferase reporter assays. We conducted an immune infiltration analysis and confirmed the bioinformatics results using immunofluorescence staining. Single-sample gene set enrichment analysis (ssGSEA) was used to analyze the metabolic mechanisms.

**Results:**

miR-182-5p-*CHL1*/*MITF* and miR-338-3p-*NOVA1* interactions were identified using in silico analysis. The qPCR results showed apparent dysregulation of these miRNA-mRNA axes in DFU. The dual-luciferase reporter assay confirmed that miR-182-5p targeted *CHL1* and *MITF*, and miR-338-3p targeted *NOVA1.* We conducted an immune infiltration analysis and observed that key genes correlated with decreased infiltration of M1 macrophages and resting mast cells in DFU. Immunofluorescence staining verified the co-localization of *CHL1* and tryptase*,* while *MITF* and CD68 showed weak positive correlations. Metabolic pathways related to these three genes were identified using ssGSEA.

**Conclusions:**

In summary, the miR-182-5p-*CHL1*/*MITF* and miR-338-3p-*NOVA1* pathway interactions and decreased infiltration of M1 macrophages and resting mast cells may provide novel clues to the pathogenesis of DFU.

**Trial registration:**

The clinical trial included in this study was registered in the Chinese Clinical Trial Registry (ChiCTR2200066660) on December 13, 2022.

**Supplementary Information:**

The online version contains supplementary material available at 10.1186/s12920-023-01741-2.

## Background

Diabetic foot ulcers (DFU) are a prevalent complication of diabetes mellitus with startling incidence and mortality rates and considerable healthcare expenses, and it has been reported that up to 26.1 million susceptible individuals develop DFU annually worldwide [1]. Statistics indicate that the five-year mortality for patients with DFU is approximately 42% [2]. Moreover, health spending on DFU was estimated at $79 billion in 2017 [3]. Although multidisciplinary treatment, which mainly includes glycemic control, lower limb amputations, transverse tibial transport, and moist-exposed burn ointment, has been applied to patients with DFU, long-term outcomes remain poor. Thus, it is imperative to identify the pathogenesis of DFU, which will be of great significance for further studies on targeted therapies.

miRNAs are endogenous small RNA molecules of approximately 20–24 nucleotides [4–7]. They modulate mRNA function mainly via the RNA-induced silencing complex and other RNA-binding proteins [8]. The most common binding sites of miRNAs are the 3' untranslated regions (3' UTRs) of target mRNA. An imbalance in the expression of miRNAs can act as an ulcerogenic factor or a suppressor of DFU [9]. For example, decreased miR-146a expression impairs neurovascular remodeling in diabetic mice [10]. The upregulation of miR-31 causes endothelial malfunction in diabetic endothelial progenitor cells [11]. miR-132 effectively promotes wound closure in db/db mice [12]. However, few studies have focused on the comprehensive miRNA-mRNA regulatory network associated with DFU pathogenesis. Hence, a reliable approach is urgently needed to explore critical miRNA-mRNA networks.

Open, broad-scale, and non-cancer omics data, such as the GEO database (http://www.ncbi.nlm.nih.gov/geo), provide clinical information and molecular data on various patients, which are useful for finding candidate miRNA-mRNA pairs [13–15]. The widespread use of high-throughput RNA sequencing has promoted the development of green, noninvasive, accurate, and high-efficiency bioinformatics tools for screening, diagnosing, evaluating, and monitoring high-risk patients [16–18]. Data processing is commonly performed using R software, bioconductor packages, and online websites. The combination of high-throughput technologies and in silico analyses is a convenient way to pursue novel mechanisms and therapeutic strategies for diseases.

**Fig. 1:**
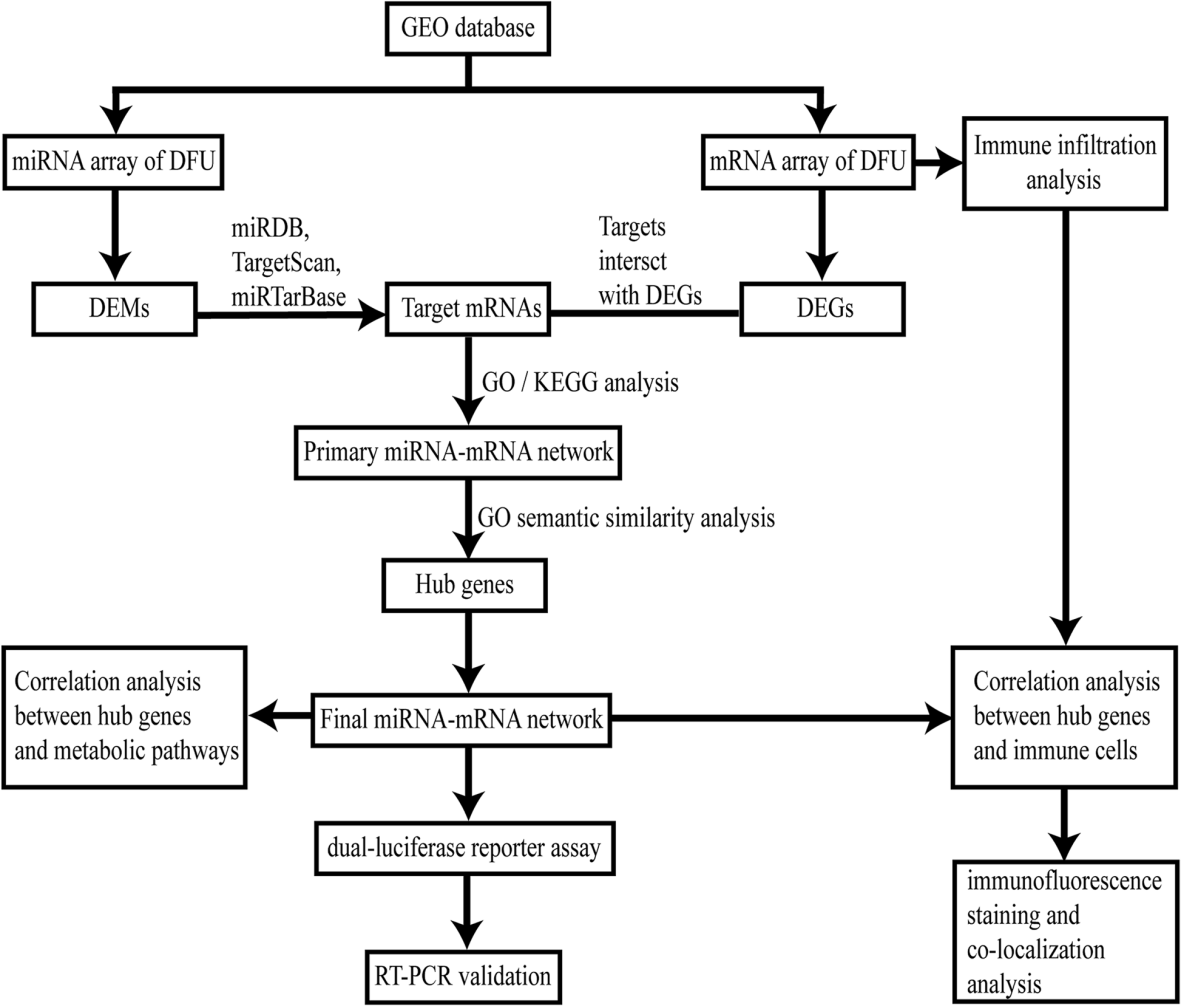
Flowchart of the study

In this study, we observed that the miR-182-5p/close homolog of L1 protein (*CHL1)*/melanocyte inducing transcription factor (*MITF)* and miR-338-3p/neuro-oncological ventral antigen 1 (*NOVA1*) networks were related to the onset of DFU. Differential expression analysis in subgroups was used to obtain the differentially expressed genes (DEGs) and differentially expressed miRNAs (DEMs) and identify their interactions. Overlaps were further screened using Gene Ontology (GO) semantic similarity analysis. We performed dual-luciferase reporter and qPCR assays to evaluate the expression levels of hub mRNAs and miRNAs. Functional enrichment analysis was performed to determine the biological significance of DEGs in the occurrence of DFU. The results showed that DEGs were mainly enriched in immune regulation and metabolic processes. Thus, we conducted immune infiltration and ssGSEA analyses to study the potential functional roles of the hub genes in DFU. Furthermore, we verified the relationship between hub genes and immune cells using immunofluorescence staining and co-localization analysis. A flowchart of the study is shown in Fig. 1.

## Methods

### Online dataset analysis

The GEO database was used to screen the miRNA and mRNA expression profiles of DFU. Only datasets that met the following standards were included: (1) expression profiling obtained from microarray or next-generation sequencing; (2) skin tissue at the edge of wounds of patients with DFU or non-DFU (NDF) individuals; and (3) at least six tissue samples in the dataset. Two eligible datasets, GSE68184 and GSE80178, were used. The GSE68184 dataset is a miRNA array that includes three DFU and three NDF samples. The GSE80178 dataset is an mRNA array constructed using nine DFU and three NDF samples. The details of these data are represented in Table S1 (Additional File 1).

### Screening of DEMs and DEGs

The “Limma” package (https://bioconductor.org/packages/release/bioc/html/limma.html) was used to output DEMs and DEGs by comparing the expression matrices between the subgroups. In this study, we used the R package to identify DEMs and DEGs between individuals with and without DFU. To control the error detection rate, a *P* value < 0.05 and |log2(fold change)| > 1 were considered as the cut-off criteria. The “ggplot2” package (https://www.rdocumentation.org/packages/ggplot2/versions/3.4.3) was used for visual analysis in R software.

### Functional enrichment analysis and protein-protein interaction (PPI) network establishment

Metascape (http://metascape.org/gp/index.html) is a user-friendly, free online database for the functional annotation and visualization of DEGs. To unveil the biological meaning of DEGs in the occurrence of DFU, we used metascape to perform GO and Kyoto Encyclopedia of Genes and Genomes (KEGG) enrichment analyses. Terms were considered significant with a *P* value ≤0.01, a min overlap ≥3, and an enrichment factor > 1.5. To further explore the connections between the DEGs, a PPI network for DEGs was constructed using the STRING (https://string-db.org) database. The results were visualized using Cytoscape (version 3.6.1) (https://cytoscape.org/release_notes_3_6_1.html).

### Prediction of DEM target genes

Three web-based resources, miRDB (http://mirdb.org/), miRTarBase (http://mirtarbase.cuhk.edu.cn/), and TargetScan (http://www.targetscan.org), were combined to identify complementary sites between DEMs and the mRNA of target genes. To guarantee the accuracy of the figures, commonly identified targets intersecting the DEGs and overlaps were selected as final targets. Cytoscape was used to present miRNA-mRNA interactions.

### Screening of hub genes

The “GOSemSim” package (https://bioconductor.org/packages/release/bioc/html/GOSemSim.html) was used in R to identify the hub genes contributing to the formation of DFU. From the perspective of GO topology, the functional similarity of genes was defined as the geometric mean of three ontologies: molecular function, cellular components, and biological pathways. We calculated the average and selected the top three hub genes to measure the GO semantic similarity of the target genes. The values were visualized in a boxplot and a raincloud graph.

### Immune infiltrate levels and expression analysis of hub genes

The CIBERSORT algorithm (https://cibersortx.stanford.edu/) was used to identify DFU-infiltrating immune cells in wound tissue. The gene expression matrix data was submitted to CIBERSORT, and the proportion of 22 types of infiltrating immune cells in individuals with DFU and their counterparts was obtained. The “vioplot” package (https://www.rdocumentation.org/packages/vioplot/versions/0.4.0/topics/vioplot) generated violin diagrams showing infiltration differences among immune cells. The “corrplot” package (https://www.rdocumentation.org/packages/corrplot/versions/0.92) was used to generate a correlation heatmap to exhibit the infiltration correlations of the 22 types of immune cells. DFU-infiltrating and DFU-related immune cells were identified. Spearman’s rank correlation analysis was performed to investigate the association between hub gene expression and the degree of immune cell infiltration. The relationships were demonstrated via a correlation heatmap generated using the “ggstatsplot” package (https://www.rdocumentation.org/packages/ggstatsplot/versions/0.12.0).

### ssGSEA and expression analysis of hub genes

ssGSEA software (version 10.0.8) (https://gsea-msigdb.github.io/ssGSEA-gpmodule/v10/), a complement to GESA, was used to analyze the metabolic mechanisms by which the hub genes might affect the onset of DFU. Datasets related to KEGG pathways were first predefined using the molecular signature database on the GSEA website (http://software.broadinstitute.org/gsea/msigdb).%20Then). ssGSEA pathway analysis was performed using the “GSVA” package (https://bioconductor.org/packages/3.17/bioc/html/GSVA.html). The selection criteria were set as a *P* value < 0.05 and a |score| > 1. The connection between essential genes and the activity of the selected pathways was investigated using Spearman’s rank correlation analysis. The results are displayed using a correlation heatmap.

### Dual-luciferase reporter assay

A dual-luciferase reporter assay was performed to verify the target relationships between hub miRNAs and genes. The *CHL1, MITF,* and *NOVA1* constructs containing wild-type (WT) or mutated (MUT) binding site vectors of mRNA 3' UTR were synthesized by GenePharma (Suzhou, China). Oligonucleotides for the miR-182-5p mimic, miR-338-3p mimic, and negative control (NC) were purchased from GenePharma. miRNA mimic and NC sequences are shown in Table S2 (Additional File 1). The 293 T cell lines were purchased from iCell (Shanghai, China). And the 293 T cells were cultured in high glucose Dulbecco’s Modified Eagle Medium (DMEM)(#11,965,126, Gibco, USA) supplemented with 10% fetal bovine serum (#16140071, Gibco, USA) and 1% penicillin and streptomycin (#P1400, Solarbio, China). The 293 T cells were cultured in a 24-well plate at 37 °C in a 5% humidified environment of CO2 and 95% air for 24 h before transfection. The *CHL1, MITF,* and *NOVA1* vectors were co-transfected with the corresponding miRNA mimic or NC into 293 T cells. After 48 h of transfection, firefly and Renilla luciferase activities were measured using a dual-luciferase kit (#E2920, Promega, USA) according to the manufacturer’s protocol.

### Tissue collection

Full-thickness skin tissues were obtained from patients receiving standard care at the Affiliated Hospital of Youjiang Medical University for Nationalities. The study protocols were approved by the Medical Ethics Committee of Youjiang Medical University for Nationalities (approval number 2018051501). All patients provided informed consent for the acquisition and use of discarded tissue. Our study included 17 patients with NDF who underwent foot surgery for acute lower-extremity trauma (nine genes and nine miRNAs) and 14 patients with DFU who underwent surgical resection of the ulcer (nine genes and nine miRNAs). We used samples from different patients to study gene and miRNA expression because some samples were tiny enough to study mRNA and miRNA simultaneously. The inclusion criteria for the DFU group were as follows: (1) age 30–65 years; (2) history of refractory DFU for > 1 month; (3) ankle-brachial index (ABI) was 0.5–0.9; and (4) Wagner grade was 2–3. The inclusion criteria for the control group were: (1) age 30–65 years; (2) no history of diabetes; (3) contusion or laceration of the lower limb within 1 week; and (4) ABI ≥0.9. Patients with any condition, such as severe necrotic tissue or osteomyelitis, or taking medications that could affect wound healing, were excluded from the study. The sample information is included in Table S3 (Additional File 1). Discarded skin samples from peri-wound tissues were collected for qPCR validation.

### Quantification of the expression of key genes and miRNAs

qPCR validated hub genes and miRNAs identified in the in silico analyses. Wound tissues from nine DFU samples and nine normal controls were collected to extract total RNA using the RNAsimple Total RNA Kit (#DP419, Tiangen, China) and then reverse-transcribed into cDNA using the FastKing RT Kit (#KR116, Tiangen, China) according to the manufacturer’s instructions. qPCR was performed on a LightCycler96 System (Roche) using SuperReal PreMix Color (#FP215, Tiangen, China). miRNAs were extracted from nine DFU samples and nine normal controls using the miRcute miRNA Isolation Kit (#DP501, Tiangen, China), reverse-transcribed, and amplified using the All-in-One miRNA qPCR Detection Kit (#QP010, Genecopoeia Co., Ltd., China). The primers were synthesized by Genecopoeia Co., Ltd. (Additional File 1: Table S4). The expression of miRNAs and targets was analyzed using the 2^-ΔΔCt^ method relative to U48 and β-actin, respectively.

### Immunofluorescence and confocal analysis

Formalin-fixed paraffin-embedded 3-μm sections were dewaxed in xylene and hydrated in ethanol. The sections were subjected to heat-mediated antigen retrieval and permeabilized in 0.2% Triton X-100 (#P1080, Solarbio, China) before blocking in PBS containing 5% goat serum (#SL038, Solarbio, China) for 1 h. The sections were then incubated with primary antibodies, either CHL1/resting mast cell (MC) marker (Tryptase), MITF/M1 macrophage marker (CD68), or NOVA1/activated NK cell marker (CD161), overnight at 4 °C. The sections were rinsed with PBS and incubated with FITC 488 and Alexa Flour 594 conjugated secondary antibodies (Additional File 1: Table S5) for 1 h at room temperature. The sections were washed in PBS, incubated with DAPI for 7 min, and mounted with an antifade mounting medium (#S2100, Solarbio, China). Confocal images were obtained using a laser-scanning confocal microscope (Leica, Germany). Quantitative co-localization analyses of the three hub genes and immune cells were performed using ImageJ and the Co-localization Finder plug-in to determine Pearson’s correlation and Mander’s coefficient.

### Statistical analysis

Each sample was analyzed in triplicate, and the statistical significance of the results was analyzed using GraphPad Prism Software (Version 8). The figures were represented as mean ± standard error. The differences between the two groups were examined using an unpaired two-tailed 𝑡 test.

## Results

### In silico analysis identified putative DEMs and DEGs

**Fig. 2:**
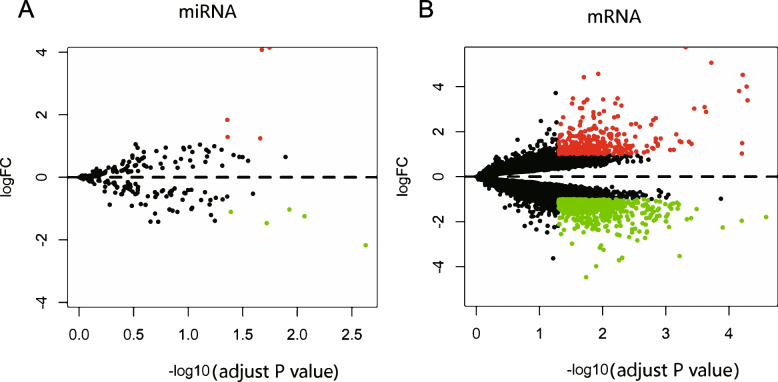
Diabetic foot ulcer (DFU) and non-DFU (NDF) differentially expressed miRNAs (DEMs) and genes (DEGs). Each point represents a gene, with red indicating significantly upregulated genes, green indicating downregulated genes, and black representing non-significant genes. (**A**) Five upregulated and five downregulated DEMs (*P* < 0.05 and |log2(fold change) | > 1). (**B**) 348 upregulated and 678 downregulated genes (*P* < 0.05 and |log2(fold change) | > 1)

To identify DEMs and DEGs between patients with and without DFU, the “R-limma” analytic method was used. The results showed five upregulated (miR-31-5p, miR-31-3p, miR-1248, miR-182-5p, and miR-27a-5p) and five downregulated DEMs (miR-338-3p, miR-136-5p, miR-100-5p, miR-136-3p, and miR-199b-5p) (Fig. 2A). Moreover, we screened 1026 DEGs, including 348 upregulated and 678 downregulated genes (Fig. 2B). Primary DEMs and DEGs were used to establish the miRNA-mRNA axis.

### Construction of a miRNA-mRNA network for DFU

**Fig. 3:**
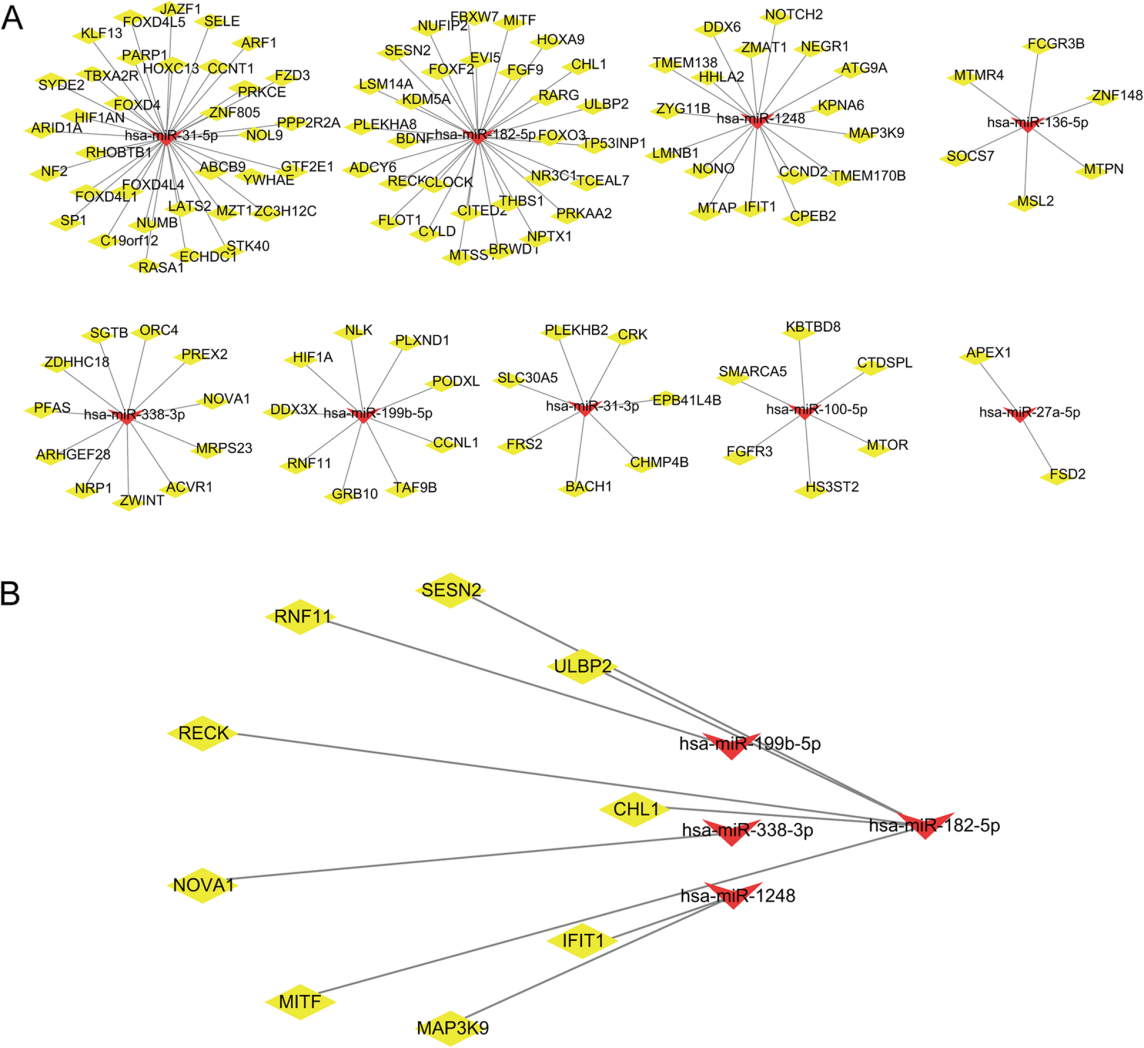
Predicted miRNA-mRNA regulatory networks. **A** Predicted target genes (yellow diamonds) of DEMs (red arrow). For one miRNA, there were no targets detected. **B** The nine final targets obtained after intersecting 122 predicted target genes with 1026 DEGs

**Fig. 4:**
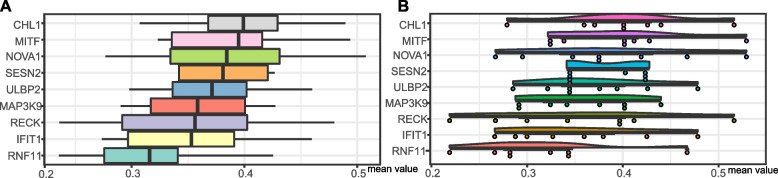
Hub genes of miRNA-mRNA regulatory axes. The distributions of functional similarities of the nine final targets visualized in a (**A**) box plot and (**B**) raincloud graph. The top three were considered hub genes

To construct a reliable miRNA-mRNA network for the pathogenesis of DFU, TargetScan, miRDB, and miRTarBase were used to predict targets. We successfully identified 122 target genes and excluded one miRNA with no targets (Fig. 3A). After quality control, we identified nine final targets (Fig. 3B), including *CHL1*, *IFIT1*, *MAP3K9*, *MITF*, *NOVA1*, *RECK*, *RNF11*, *SESN2,* and *ULBP2,* and Cytoscape was used to present miRNA-mRNA interactions. We explored the hub genes of these common targets using GO semantic similarity analysis. The top three genes were *CHL1*, *MITF,* and *NOVA1* (Fig. 4A, B). *CHL1* and *MITF* were negatively regulated by miR-182-5p, and *NOVA1* was negatively regulated by miR-338-3p. Therefore, we identified two miRNA-mRNA regulatory networks that may be associated with the occurrence of DFU.

### Dual-luciferase reporter assay for validation of miRNA-mRNA regulatory networks

**Fig. 5:**
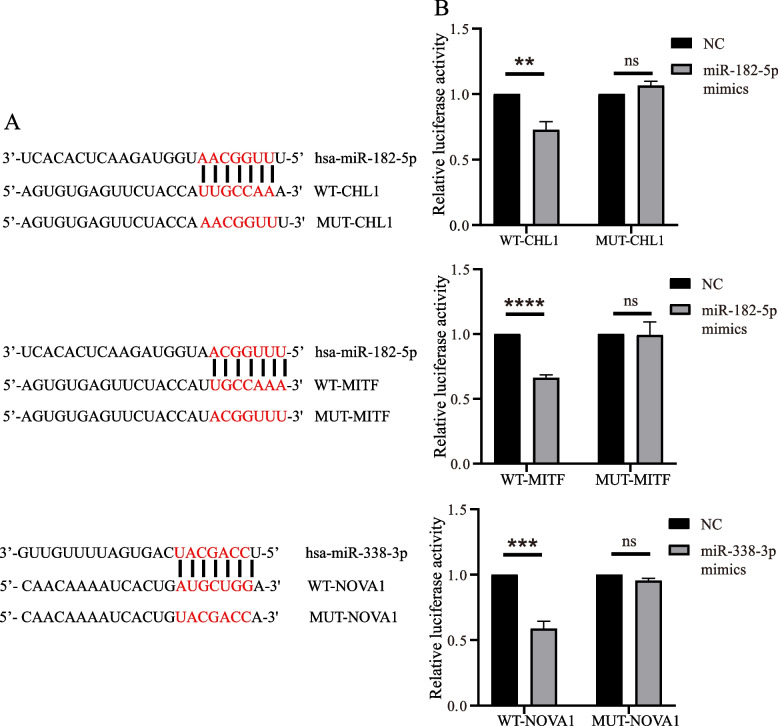
Dual-luciferase reporter assay for validation of miRNA-mRNA regulatory networks. **A** The three predicted binding sites between miRNA-182-5p or miRNA-338-3p and the 3' untranslated region (3' UTR) of the corresponding genes. **B** The relative luciferase activity in 293 T cells co-transfected with wild-type (WT) or mutant (MUT) binding site vectors of *CHL1*, *MITF,* and *NOVA1* 3' UTR in the presence of miRNA mimics or a negative control (NC). *𝑃< 0.05, **𝑃< 0.01, ***𝑃< 0.001, ****𝑃< 0.0001

To determine whether *CHL1* and *MITF* were direct targets of miR-182-5p and *NOVA1* was a direct target of miR-338-3p, a dual-luciferase reporter assay was performed using *CHL1*, *MITF,* and *NOVA1* reporter constructs. Three candidate binding sites were observed between the 3' UTR of hub genes and miRNAs (Fig. 5A). The relative luciferase activity was significantly suppressed in 293 T cells co-transfected with the WT 3' UTR binding site vectors and miRNA mimics compared with cells co-transfected with NC. However, no significant difference was observed in 293 T cells co-transfected with the MUT 3' UTR binding site vectors and miRNA mimics or NC (Fig. 5B). These results suggested that *CHL1* and *MITF* were direct target genes of miR-182-5p, and *NOVA1* was a direct target gene of miR-338-3p.

### Expression of hub miRNAs and genes in DFU wound tissues

**Fig. 6:**
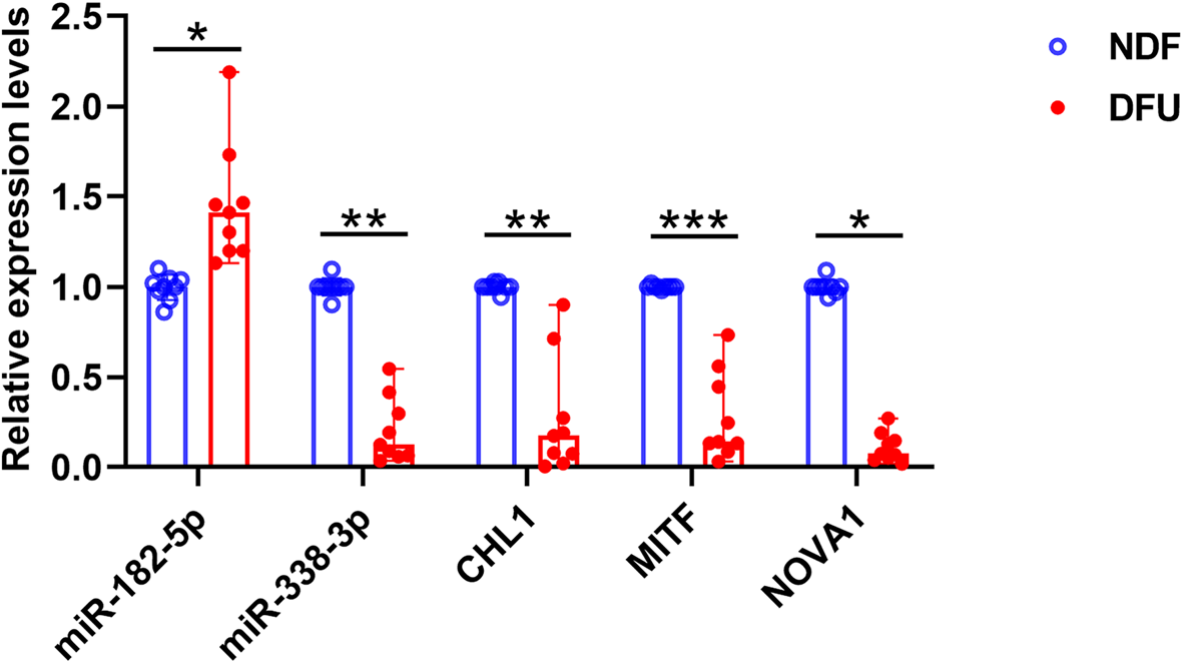
Clinical significance of miR-182-5p, miR-338-3p, *CHL1*, *MITF,* and *NOVA1*. The relative expression levels of miR-182-5p, miR-338-3p, *CHL1*, *MITF,* and *NOVA1*. Nine samples were used to study gene expression, and nine were used to study the expression of miRNA. All experiments were carried out in triplicate. *𝑃< 0.05, **𝑃< 0.01, ***𝑃< 0.001, ****𝑃< 0.0001

To confirm the hub miRNAs identified using bioinformatics tools, we performed qPCR assays on the two miRNAs and three genes in the wound tissues of patients with and without DFU. We observed that miR-182-5p was significantly upregulated and miR-338-3p was significantly downregulated in DFU tissues compared to that in normal tissues. *CHL1*, *MITF,* and *NOVA1* were significantly downregulated in DFU compared to those in healthy controls. The results of the qPCR experiments followed those of the bioinformatics analysis (Fig. 6).

### Function correlation analyses and PPI networks

**Fig. 7:**
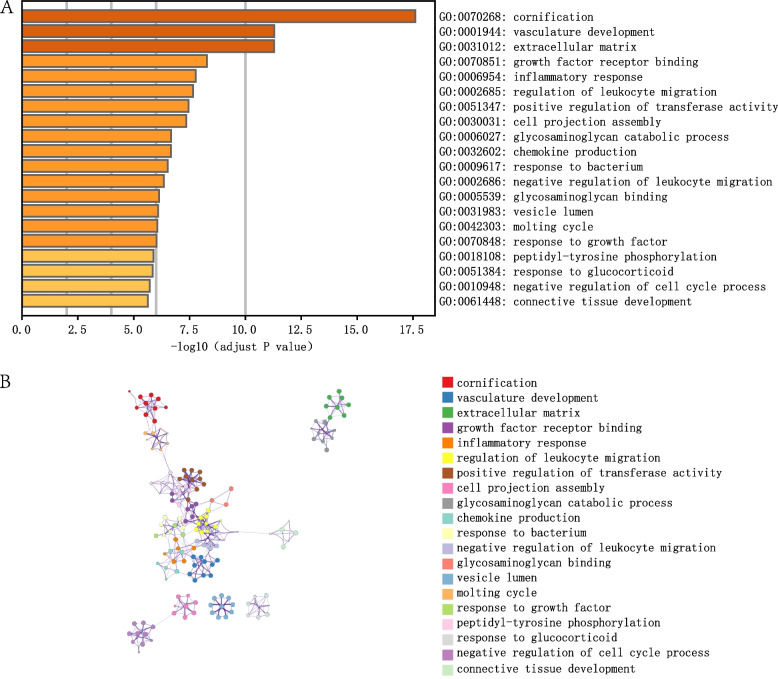
Functional enrichment analysis of DEGs. **A** Top 20 gene ontology (GO) terms enriched by DEGs, colored by *P* value. **B** Networks of GO-enriched terms. Colors indicate GO terms, and opacity indicates the P value

To reveal the biological functions of DEGs in the incidence of DFU, we performed an enrichment analysis using metascape. The DEGs were mainly associated with the inflammatory response, regulation of leukocyte migration, chemokine production, response to bacteria, and glycosaminoglycan catabolic processes (Fig. 7A, B). To analyze the potential interactions between DEGs, the STRING online tool was used to establish a PPI network (Additional File 1: Fig. S4). No direct relationships were observed among the hub genes. Overall, the functions of the DEGs are significantly correlated with immune responses and metabolic processes.

### Correlation between immune infiltration and expression of hub genes in DFU

**Fig. 8:**
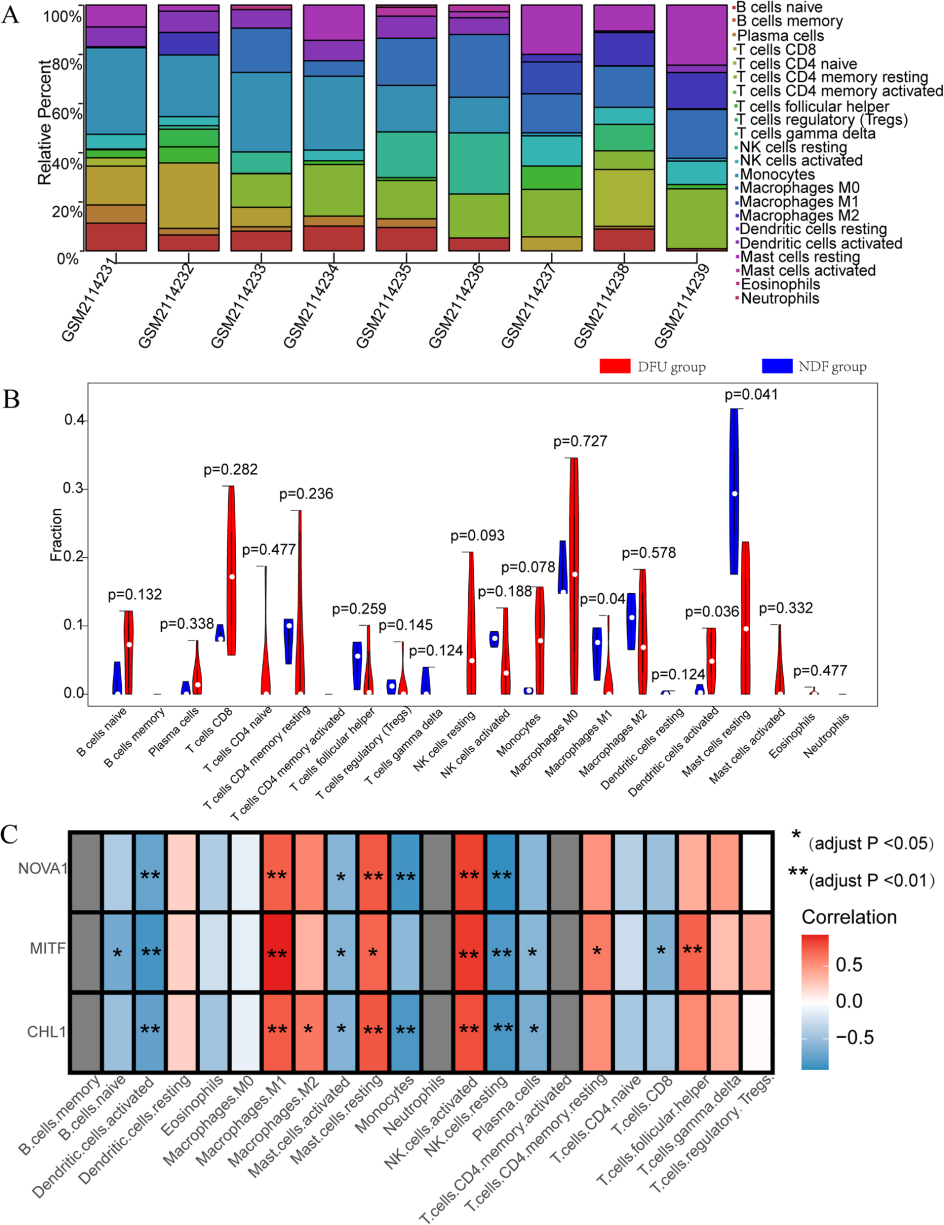
The immune infiltration results of patients with and without DFU. **A** The composition of the 22 immune cell types in each patient sample of DFU and normal tissues. **B** The infiltration difference of immune cells between the DFU (red) and NDF (blue) groups. **C** The correlation among infiltrating immune cells and CHL1, MITF, and NOVA1. Red indicates a positive correlation, and blue indicates a negative correlation. *𝑃< 0.05, **𝑃< 0.01, ***𝑃< 0.001

To identify DFU-infiltrating immune cells, CIBERSORT tools were used. The bar plot showed that monocytes and CD8 + T cells were the main immune cell types that infiltrated the tissues of patients with DFU (Fig. 8A). The violin plot showed that the DFU tissue contained a higher proportion of activated dendritic cells than that in the control group. In contrast, the proportion of M1 macrophages and resting MCs was lower (Fig. 8B). The correlation heatmap showed that follicular helper T cells, monocytes, and activated NK cells were associated with these differentially infiltrated cells (Additional File 1: Fig. S1). We performed a correlation analysis to explore the association between hub genes and the 22 types of immune cells. The results indicated that the strongest positive correlations were between *CHL1* and resting MCs, *MITF* and M1 macrophages, and *NOVA1* and activated NK cells (Fig. 8C). We intersected the DFU-infiltrating immune cells, and immune cells positively correlated with hub genes. We observed that fewer infiltrating M1 macrophages and resting MCs may be related to the occurrence of DFU.

### Co-localization of CHL1 with tryptase, MITF with CD68, and NOVA1 with CD161 in DFU tissues

**Fig. 9:**
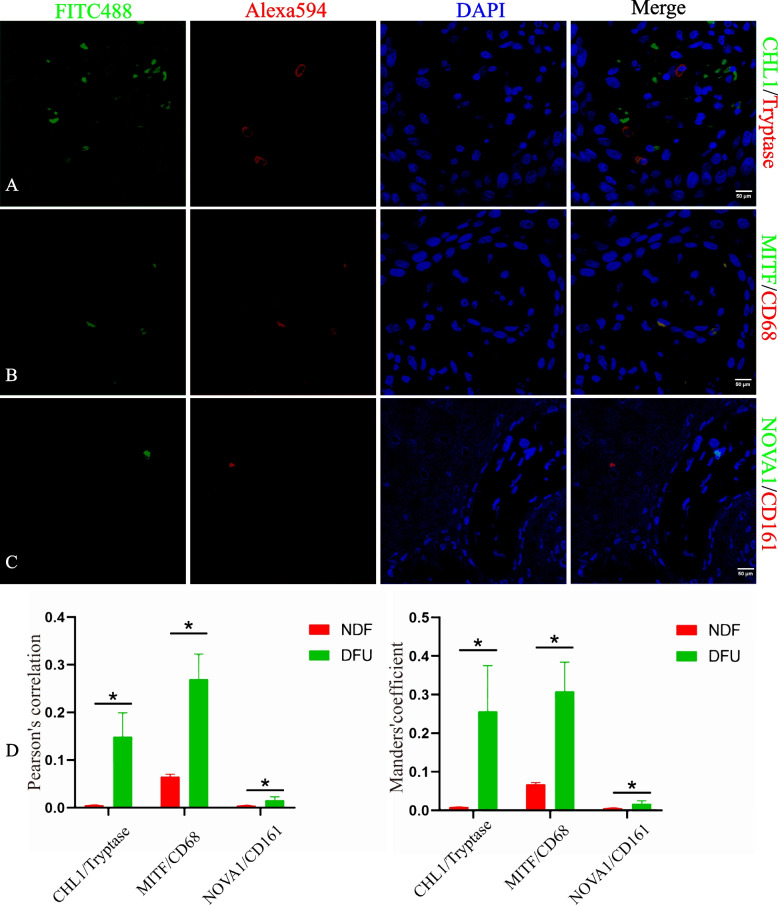
Co-localization of CHL1 with tryptase, MITF with CD68, and NOVA1 with CD161 in DFU tissues. Immunofluorescence staining using (**A**) anti-CHL1 and anti-Tryptase, **B** anti-MITF and anti-CD68, and **C** anti-NOVA1 and anti-CD161 antibodies. Tissues are shown at 80× magnification. **D** Quantification (Pearson’s correlation and Manders’ coefficient) of co-localization of hub genes with immune cell markers. Results are represented as mean ± standard error. *𝑃< 0.05, **𝑃< 0.01, ***𝑃< 0.001

Immunofluorescence staining was performed to visualize the co-localization of hub genes with immune cell types and to confirm the distribution of these proteins in DFU (Fig. 9) and NDF (Additional File 1: Fig. S3) tissues. Compared with NDF tissues, a significant weak positive co-localization of CHL1 with tryptase (Pearson’s correlation = 0.15 ± 0.05, Mander’s coefficient = 0.26 ± 0.12) was observed in DFU tissues. A significant strong positive co-localization of MITF with CD68 (Pearson’s correlation = 0.27 ± 0.05, Manders’ coefficient = 0.31 ± 0.08) was observed in DFU tissues. However, there was little co-localization of NOVA1 with CD161 (Pearson’s correlation = 0.02 ± 0.01, Mander’s coefficient = 0.02 ± 0.01) in DFU tissues. It was also statistically significant. The correlation between CHL1 and MITF with tryptase and CD68, respectively, suggests a potential interaction between CHL1 and resting MCs and MITF and M1 macrophages in DFU tissues.

### Relationship between metabolic pathways and expression of hub genes in DFU

To elucidate the underlying mechanism of DFU onset, ssGSEA analysis was implemented. The analysis identified 17 metabolism-related pathways that met eligibility, which were further explored for correlation between hub genes and the pathways. We observed that *CHL1* was most positively correlated with the glycosphingolipid biosynthesis ganglio series pathway and negatively correlated with the PPAR signaling pathway. *MITF* was most positively associated with the ether lipid metabolism pathway and most negatively associated with the glycosphingolipid biosynthesis lacto and neolacto series pathways. *NOVA1* had the strongest positive correlation with the glycosphingolipid biosynthesis ganglio series pathway and a negative correlation with the glycosphingolipid biosynthesis lacto and neolacto series pathway (Additional File 1: Fig. S2). The results of this analysis showed that the glycosphingolipid biosynthesis ganglio series and glycosphingolipid biosynthesis lacto pathways could be the most relevant biological pathways involved in DFU pathogenesis.

## Discussion

DFU has become a concern for many patients with diabetes worldwide. The miRNA-mRNA network is linked to the onset of various human diseases, including DFU. However, studies on the miRNA-mRNA regulatory network in DFU remain insufficient. In this study, miRNA-mRNA regulatory axes containing two miRNAs and three mRNAs were identified through integrated bioinformatics analyses. Enrichment analysis revealed that the functions of DEGs were primarily concentrated in immune cells and metabolic cascades. Therefore, we performed immune infiltration and ssGSEA analyses. Moreover, we explored the association between hub genes and immune cell infiltration or metabolic pathways in DFU. Finally, the three key regulatory interactions were verified using dual-luciferase reporter and qPCR assays. The relationship between these three genes and immune cells was confirmed using immunofluorescence. Thus, we concluded that DFU pathogenesis may be related to the miR-182-5p-*CHL1/MITF* and miR-338-3p-*NOVA1* networks and dysregulated resting MCs and M1 macrophages.

The pathogenesis of DFU is complex and not fully understood; peripheral neuropathy, vasculopathy, and infections are considered the main causes of DFU. Systemic factors, including continuous hyperglycemia and inflammatory and immune responses, are involved in the pathophysiology of the disease [19–21]. Previous bioinformatics analyses have mainly focused on miRNAs in several diseases. For example, miR15b and miR200b suppressed VEGF and VEGF-R2 mRNAs, upregulated and inhibited impaired angiogenesis in an in vivo model of diabetic wounds. Anti-miR15b and anti-miR200b treatments improve wound repair [22]. These findings indicate that anti-miR therapy has potential applications in diabetes-related wounds. However, a miRNA-mRNA network may be better for exploring the mechanism of DFU and for further targeted treatment. Dawidowska et al. [23] reported that miR-363-3p-*LATS2* might be a prospective clinical marker for T-cell acute lymphoblastic leukemia. Herein, we report a new method for molecular mechanism identification by constructing a holistic mRNA-miRNA regulatory network.

In this study, two regulatory networks, miR-182-5p-CHL1/MITF, and miR-338-3p-NOVA1, were associated with DFU, which may provide new insights into therapeutic strategies for DFU. miR-182-5p has been proposed to be associated with dysglycemia and could potentially predict diabetes [24]. Similarly, miR-338-3p may act as an indicator of type 2 diabetes [25]. *CHL1* encodes a neural adhesion molecule that promotes clathrin-mediated vesicle endocytosis in synapses. Moreover, *CHL1* regulates insulin secretion in INS-1 cells [26]. Therefore, *CHL1* may be involved in the occurrence of DFU via neurotransmitter-dependent insulin secretion. *MITF* is a member of the *MITF* family [27], and it is reported that *MITF* controls β cell function depending on the interplay between *MITF* and paired box 6 (*Pax6*) [28]. Therefore, *MITF* may alter β cell function in patients with DFU. *NOVA* is a family of alternative splicing factors, including *NOVA1* and *NOVA2* [29]. *NOVA1* regulates insulin release [30] and fibroblast proliferation in patients with DFU [31]. Hence, *NOVA1* is a prospective target for DFU therapeutics due to its local and systemic modulation of the disease. In this study, qPCR confirmed that miR-182-5p expression were significantly increased while *CHL1*, *MITF*, *NOVA1,* and miR-338-3p expression was significantly decreased in DFU tissues compared to those in normal tissues. The dual-luciferase reporter assay revealed that miR-182-5p targeted *CHL1/MITF* 3' UTR and miR-338-3p targeted *NOVA1* 3' UTR in 293 T cells. In brief, these results indicate that both miR-182-5p-*CHL1*/*MITF* and miR-338-3p-*NOVA1* interactions might play a role in the pathogenesis of DFU, and miR-338-3p-*NOVA1* crosstalk, which provides a theoretical basis for initial therapeutic attempts to target these networks.

Through GO analysis, we observed that the functions of the hub genes were mainly involved in immune cells in DFU. Likely, the dysregulation of infiltrating immune cells and their interaction with hub genes play a role in initiating DFU. The results of our study indicated that *CHL1, MITF,* and *NOVA1* might have reduced the immune cell infiltrate abundance of DFU, particularly M1 macrophages and resting MCs. Previous investigations have revealed a decreased infiltration of M1 macrophages into chronic diabetic wounds [32]. Another study demonstrated that M1 macrophages clean the wound from bacteria, dead cells, and foreign debris in DFU by secreting nitric oxide and reactive oxygen species [33]. Furthermore, M1 macrophages may promote immune responses in resting MCs. A few studies have reported that insufficient resting MCs before wounding and the inability to mount acute degranulation after wounding collectively cause the formation and development of DFU [34]. In this study, decreased infiltration of M1 macrophages and resting MCs in DFU tissues was positively correlated with the three hub genes. However, the roles of *CHL1, MITF,* and *NOVA1* in the immune mechanisms of DFU are unknown. *CHL1-*deficiency reduces the recruitment of macrophages and impairs the balance of Th17/Treg cells in mice with inflammatory bowel disease [35]. *MITF* is a potential immune response gene associated with the pathogenesis of IgE/MCs-mediated anaphylaxis [36]. *NOVA1* suppression may affect the prognosis of gastric cancer by altering the proportion of macrophages and T cells in the tumor microenvironment [37]. This study demonstrated a positive correlation between *CHL1,* resting MCs, *MITF* and M1 macrophages, and *NOVA1* and activated NK cells. Altogether, *CHL1, MITF,* and *NOVA1*, especially *CHL1* and *MITF*, may be involved in the immune dysregulation of DFU and may serve as predictors of immune infiltration in DFU.

## Conclusions

Our study identified effective miRNA-mRNA regulatory pairs for DFU and offered insights into the landscape of immune cells associated with DFU. miR-182-5p-*CHL1/MITF* and miR-338-3p-*NOVA1* networks, along with resting MCs and M1 macrophages, might participate in the occurrence of DFU. Therefore, this study provides a new perspective on the immune cellular-molecular mechanisms underlying DFU and potential treatment strategies.

### Supplementary Information

**Additional file 1:** **Table S1.** Basic information of chosen datasets. **Table S2.** Sequence of miR-182-5p mimic, miR-338-3p mimic and negative control. **Table S3.** Sample information of different patients. Aca, Acarbose; C, China; DFU, Diabetic Foot Ulcer; DK, Diabetic Ketosis; DN, Diabetic Nephropathy; DPN, Diabetic Peripheral Neuropathy; DR, Diabetic Retinopathy; F, Female; H, hypertension; I, infection; INS, insulin; LEA, Lower Extremity Atherosclerosis; M, Male; Met, Metformin; NDF, non-DFU; O, osteomyelitis. **Table S4.** Primers used in qPCR test. **Table S5.** Antibodies used for the immunofluorescence study. **Figure S1.** The association with 22 types of immune cells in DFU (A) and NDF (B) tissues. Red: positive correlation; blue: negative correlation. **Figure S2.** The relationship between 17 metabolic pathways and CHL1, MITF together with NOVA1, respectively. Red: positive correlation; blue: negative correlation. (*𝑃<0.05, **𝑃<0.01, ***𝑃<0.001). **Figure S3.** Co-localization of CHL1 with tryptase, MITF with CD68, and NOVA1 with CD161 in NDF tissues. Immunofluorescence staining using (A) anti-CHL1 and anti-Tryptase, (B) anti-MITF and anti-CD68, and (C) anti-NOVA1 and anti-CD161 antibodies. Tissues are shown at 80× magnification. **Figure S4**. Protein-protein interaction (PPI) network of DEGs.

## Data Availability

Publicly available datasets were analyzed in this study. The GSE68184 and GSE80178 data are available from GEO.

## Abbreviations

3' UTR: 3' untranslated region
CHL1: Close homolog of L1 protein
DEG: Differentially expressed gene
DEM: Differentially expressed miRNA
DFU: Diabetic foot ulcers
DMEM: Dulbecco’s Modified Eagle Medium
GEO: Gene Expression Omnibus
GO: Gene Ontology
KEGG: Kyoto Encyclopedia of Genes and Genomes
MC: Mast cell
miRNA: MicroRNA
MITF: Melanocyte inducing transcription factor
MUT: Mutated-type
NC: Negative control
NDF: Non-DFU
NOVA: Neuro-oncological ventral antigen
Pax6: Paired box 6
PPI: Protein-protein interaction
ssGSEA: Single sample gene set enrichment analysis
qPCR: quantitative real-time PCR
WT: Wild-type
