# Supplementary material for: Identifying potential pathogenesis and immune infiltration in diabetic foot ulcers using bioinformatics and in vitro analyses
Source: BMC Med Genomics. 2023 Dec 1;16:313. doi: 10.1186/s12920-023-01741-2 (PMC10693102; doi:10.1186/s12920-023-01741-2)
Supplement: Supplementary file 1 — Additional file 1: Table S1. Basic information of chosen datasets. Table S2. Sequence of miR-182-5p mimic, miR-338-3p mimic and negative control. Table S3. Sample information of different patients. Aca, Acarbose; C, China; DFU, Diabetic Foot Ulcer; DK, Diabetic Ketosis; DN, Diabetic Nephropathy; DPN, Diabetic Peripheral Neuropathy; DR, Diabetic Retinopathy; F, Female; H, hypertension; I, infection; INS, insulin; LEA, Lower Extremity Atherosclerosis; M, Male; Met, Metformin; NDF, non-DFU; O, osteomyelitis. Table S4. Primers used in qPCR test. Table S5. Antibodies used for the immunofluorescence study. Figure S1. The association with 22 types of immune cells in DFU (A) and NDF (B) tissues. Red: positive correlation; blue: negative correlation. Figure S2. The relationship between 17 metabolic pathways and CHL1, MITF together with NOVA1, respectively. Red: positive correlation; blue: negative correlation. (*𝑃<0.05, **𝑃<0.01, ***𝑃<0.001). Figure S3. Co-localization of CHL1 with tryptase, MITF with CD68, and NOVA1 with CD161 in NDF tissues. Immunofluorescence staining using (A) anti-CHL1 and anti-Tryptase, (B) anti-MITF and anti-CD68, and (C) anti-NOVA1 and anti-CD161 antibodies. Tissues are shown at 80× magnification. Figure S4. Protein-protein interaction (PPI) network of DEGs. [file 12920_2023_1741_MOESM1_ESM.docx]

**Additional file 1**: **Table S1**. Basic information of chosen datasets. **Table S2**. Sequence of miR-182-5p mimic, miR-338-3p mimic and negative control. **Table S3**. Sample information of different patients. Aca, Acarbose; C, China; DFU, Diabetic Foot Ulcer; DK, Diabetic Ketosis; DN, Diabetic Nephropathy; DPN, Diabetic Peripheral Neuropathy; DR, Diabetic Retinopathy; F, Female; H, hypertension; I, infection; INS, insulin; LEA, Lower Extremity Atherosclerosis; M, Male; Met, Metformin; NDF, non-DFU; O, osteomyelitis. **Table S4**. Primers used in qPCR test. **Table S5**. Antibodies used for the immunofluorescence study. **Figure S1**. The association with 22 types of immune cells in DFU (A) and NDF (B) tissues. Red: positive correlation; blue: negative correlation. **Figure S2**. The relationship between 17 metabolic pathways and CHL1, MITF together with NOVA1, respectively. Red: positive correlation; blue: negative correlation. (*𝑃<0.05, **𝑃<0.01, ***𝑃<0.001). **Figure S3**. Co-localization of CHL1 with tryptase, MITF with CD68, and NOVA1 with CD161 in NDF tissues. Immunofluorescence staining using (A) anti-CHL1 and anti-Tryptase, (B) anti-MITF and anti-CD68, and (C) anti-NOVA1 and anti-CD161 antibodies. Tissues are shown at 80× magnification. **Figure S4**. Protein-protein interaction (PPI) network of DEGs.

Table S1 Basic information of chosen datasets

| GEO accession | Platform | Tissue (Homo sapiens) | Experiment type | Total Samples (number) | DFU Samples | NDF Samples |
| --- | --- | --- | --- | --- | --- | --- |
| GSE68184 | GPL19631, Exiqon human V3 microRNAbpanel I+II | Skin | Array | 6 | 3 | 3 |
| GSE80178 | GPL16686, Affymetrix Human Gene 2.0 ST Array | Skin | Array | 12 | 9 | 3 |

Table S2 Sequence of miR-182-5p mimic, miR-338-3p mimic and negative control

| Oligonucleotides | Sense (5'-3') | Anti-sense (5'-3') |
| --- | --- | --- |
| miR-182-5p mimic | UUUGGCAAUGGUAGAACUCACACU | UGUGAGUUCUACCAUUGCCAAAUU |
| miR-338-3p mimic | UCCAGCAUCAGUGAUUUUGUUG | ACAAAAUCACUGAUGCUGGAUU |
| miR-NC | UUCUCCGAACGUGUCACGUTT | ACGUGACACGUUCGGAGAATT |

Table S3 Sample information of different patients

| Sample | Age | Gender(M/F) | | Item | Ethnicity | | Smoking | Medications | Comorbidities |
| --- | --- | --- | --- | --- | --- | --- | --- | --- | --- |
| DFU | 53 | M | Gene, miR, IF | | | C | No | Ins | I, O, LEA |
| DFU | 59 | M | Gene, IF | | | C | No | Ins, met | I, LEA |
| DFU | 54 | M | Gene, IF | | | C | No | Ins | I, H, LEA |
| DFU | 59 | M | gene | | | C | No | Ins | I |
| DFU | 64 | M | gene | | | C | No | Ins | I, LEA |
| DFU | 54 | M | gene | | | C | No | Ins | I |
| DFU | 64 | M | gene | | | C | No | Ins | I |
| DFU | 55 | M | gene | | | C | No | Ins | I |
| DFU | 65 | M | gene | | | C | No | Ins | I, LEA |
| DFU | 54 | M | miR | | | C | No | Ins | I, H, DN, LEA |
| DFU | 49 | M | miR | | | C | Yes | Met | H, DN |
| DFU | 50 | M | miR | | | C | No | Ins | DK, DPN, LEA |
| DFU | 63 | M | miR | | | C | No | Ins | DR, DPN, LEA |
| DFU | 52 | F | miR | | | C | No | Ins, aca | H, DN, LEA |
| DFU | 63 | M | miR | | | C | No | Ins | I, O |
| DFU | 47 | M | miR | | | C | No | Ins | No |
| DFU | 59 | M | miR | | | C | No | Ins, met | O, LEA |
| NDF | 46 | M | Gene, miR | | | C | No | No | No |
| NDF | 40 | M | Gene, miR | | | C | No | No | No |
| NDF | 58 | F | Gene, miR | | | C | No | No | No |
| NDF | 56 | F | Gene, miR | | | C | No | No | No |
| NDF | 41 | M | gene | | | C | No | No | No |
| NDF | 45 | M | gene | | | C | No | No | No |
| NDF | 54 | M | gene | | | C | No | No | No |
| NDF | 33 | M | gene | | | C | No | No | No |
| NDF | 42 | M | gene | | | C | No | No | No |
| NDF | 47 | M | miR | | | C | No | No | No |
| NDF | 48 | M | miR | | | C | No | No | No |
| NDF | 51 | F | miR | | | C | No | No | No |
| NDF | 37 | M | miR | | | C | No | No | No |
| NDF | 43 | M | miR | | | C | No | No | No |

Note: Aca, Acarbose; C, China; DFU, Diabetic Foot Ulcer; DK, Diabetic Ketosis; DN, Diabetic Nephropathy; DPN, Diabetic Peripheral Neuropathy; DR, Diabetic Retinopathy; F, Female; H, hypertension; I, infection (Mild infection without tissue necrosis and osteomyelitis); INS, insulin; LEA, Lower Extremity Atherosclerosis; M, Male; Met, Metformin; NDF, non-DFU; O, osteomyelitis.

Table S4 Primers used in qPCR test

| Primer | Forward (5'-3') | Reverse (5'-3') |
| --- | --- | --- |
| Has-miR-182-5p | TTGGCAATGGTAGAACTCACACTAA |  |
| Has-miR-338-3p | CAGCATCAGTGATTTTGTTGAAA |  |
| U48 | The primer sequences are confidential. |  |
| CHL1 | CAGTGGTTGGGTACAGTGCT | CCAGCATCTTCTTCGGTGGT |
| MITF | ACCACATACAGCAAGCCCAA | GACATGGCAAGCTCAGGACT |
| NOVA1 | TACTGAGCGAGTGTGCTTGAT | GTCTGGGGTTGTAGAATGCTG |
| β-actin | CCTGGCACCCAGCACAAT | GGGCCGGACTCGTCATAC |

Table S5 Antibodies used for the immunofluorescence study

| Antibodies | Host | Dilution | Manufacturer |
| --- | --- | --- | --- |
| Anti-CHL1 | Rabbit | 1:200 | Affinity |
| Anti-Tryptase | Mouse | 1:250 | Abcam |
| Anti-MITF | Rabbit | 1:100 | Affinity |
| Anti-CD68 | Mouse | 1:100 | Bioss |
| Anti-NOVA1 | Rabbit | 1:250 | Abcam |
| Anti-CD161 | Mouse | 1:200 | Proteintech |
| Fluorescein®488 Goat anti-Rabbit IgG | Goat | 1:500 | ZSGB-BIO |
| Alexa Flour®594 Goat anti-Mouse IgG | Goat | 1:500 | ZSGB-BIO |


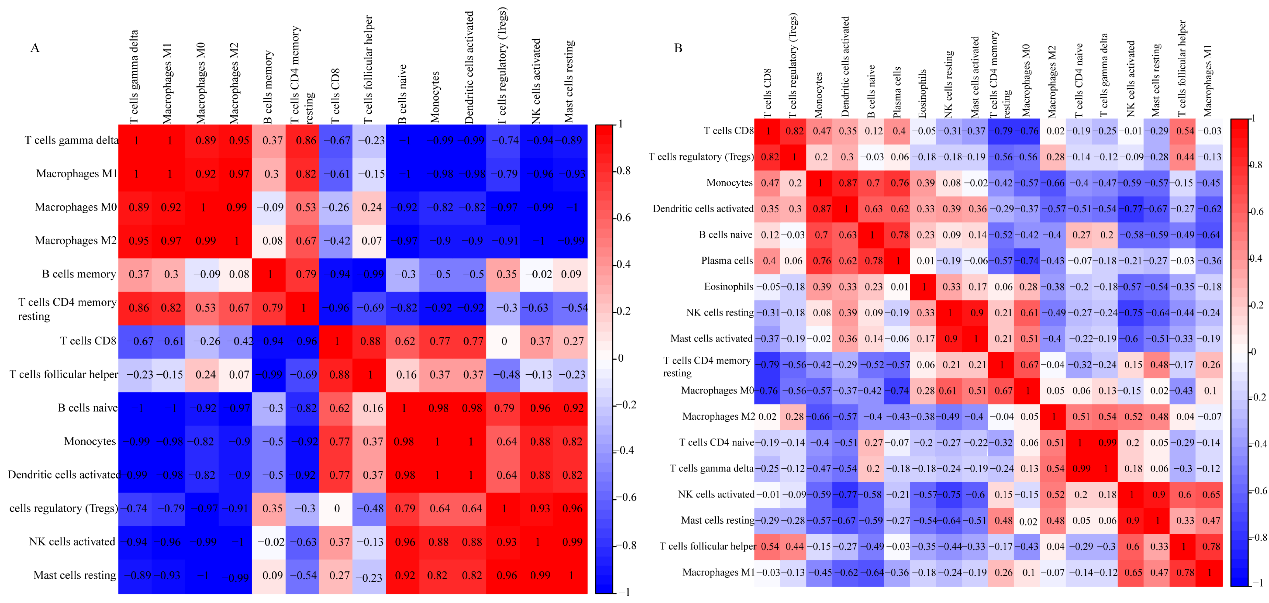


**Figure S1.** The association with 22 types of immune cells in DFU (A) and NDF (B) tissues. Red: positive correlation; blue: negative correlation.


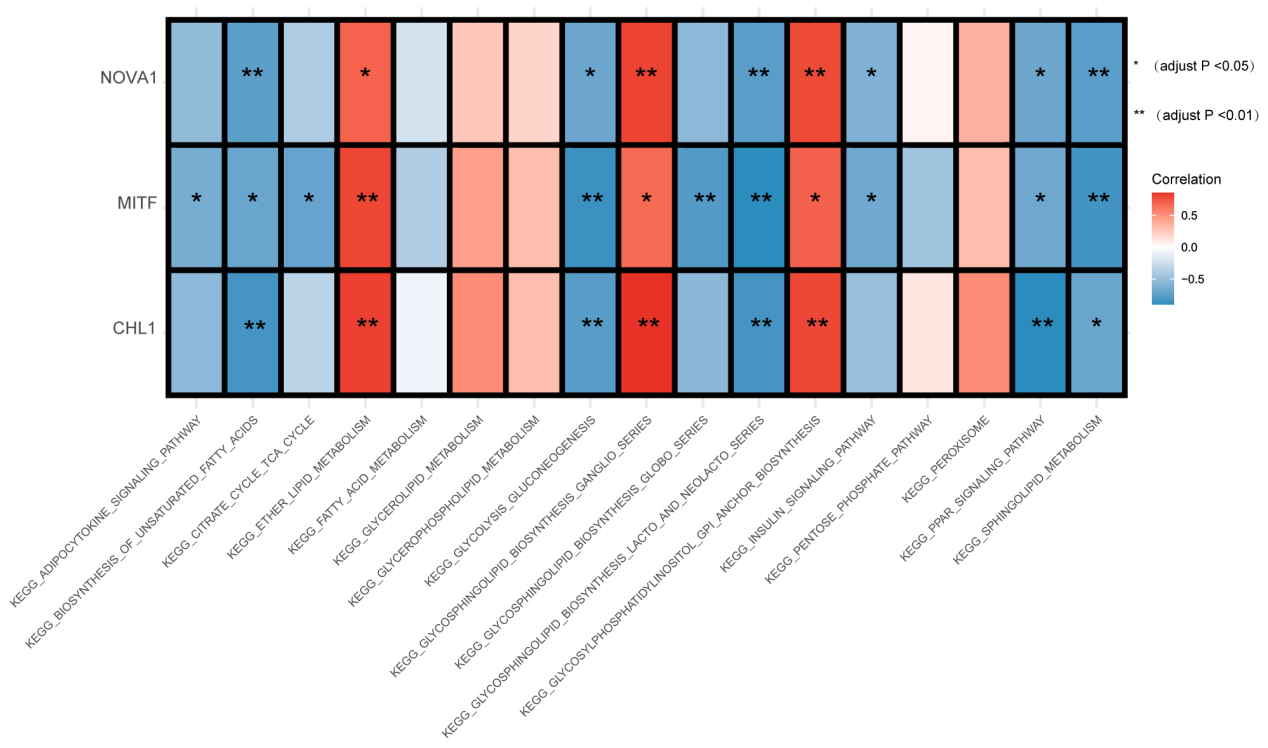


**Figure S2.** The relationship between 17 metabolic pathways and CHL1, MITF together with NOVA1, respectively. Red: positive correlation; blue: negative correlation. (*𝑃<0.05, **𝑃<0.01, ***𝑃<0.001).


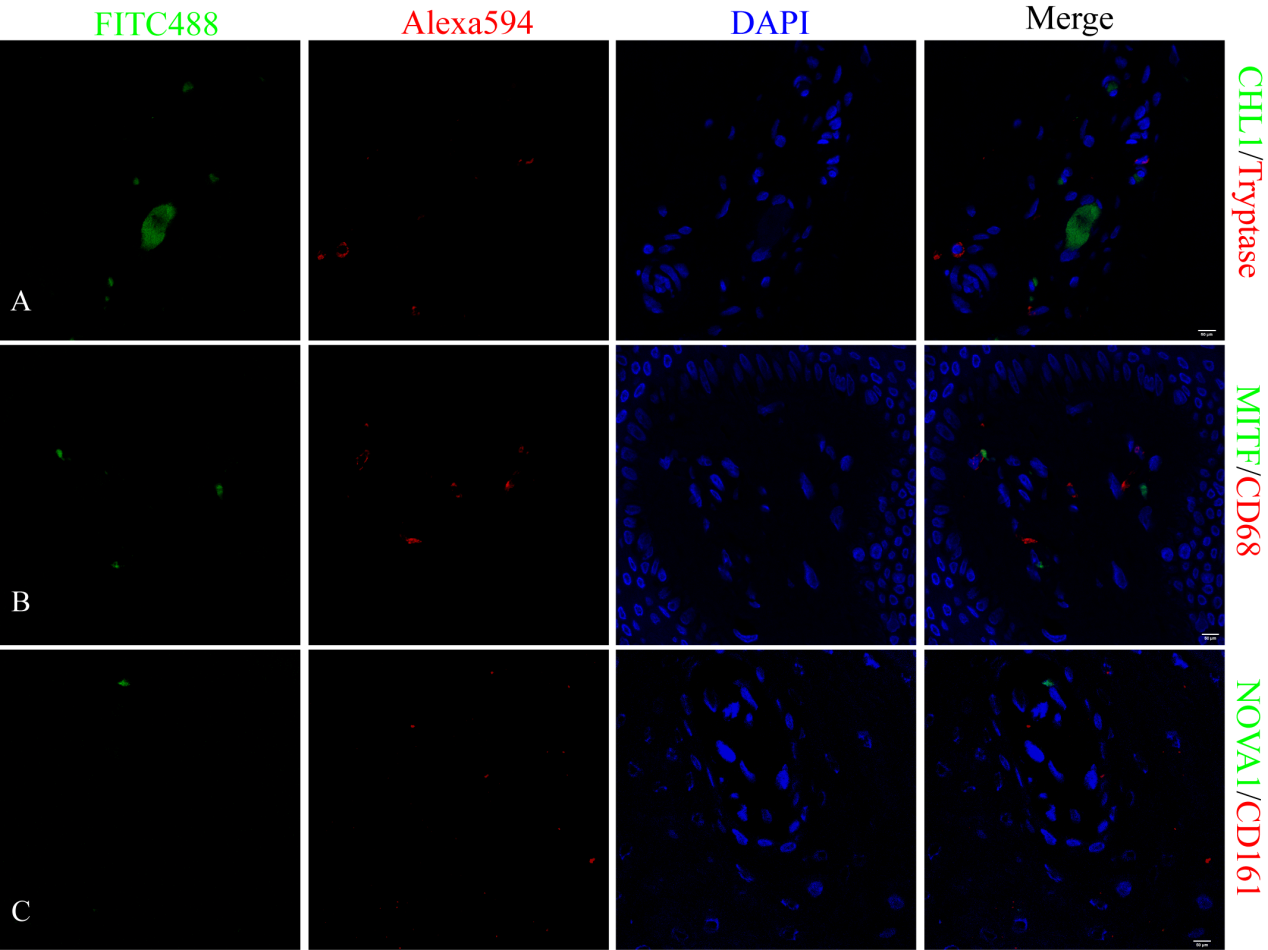


**Figure S3**. Co-localization of CHL1 with tryptase, MITF with CD68, and NOVA1 with CD161 in NDF tissues. Immunofluorescence staining using (A) anti-CHL1 and anti-Tryptase, (B) anti-MITF and anti-CD68, and (C) anti-NOVA1 and anti-CD161 antibodies. Tissues are shown at 80× magnification.


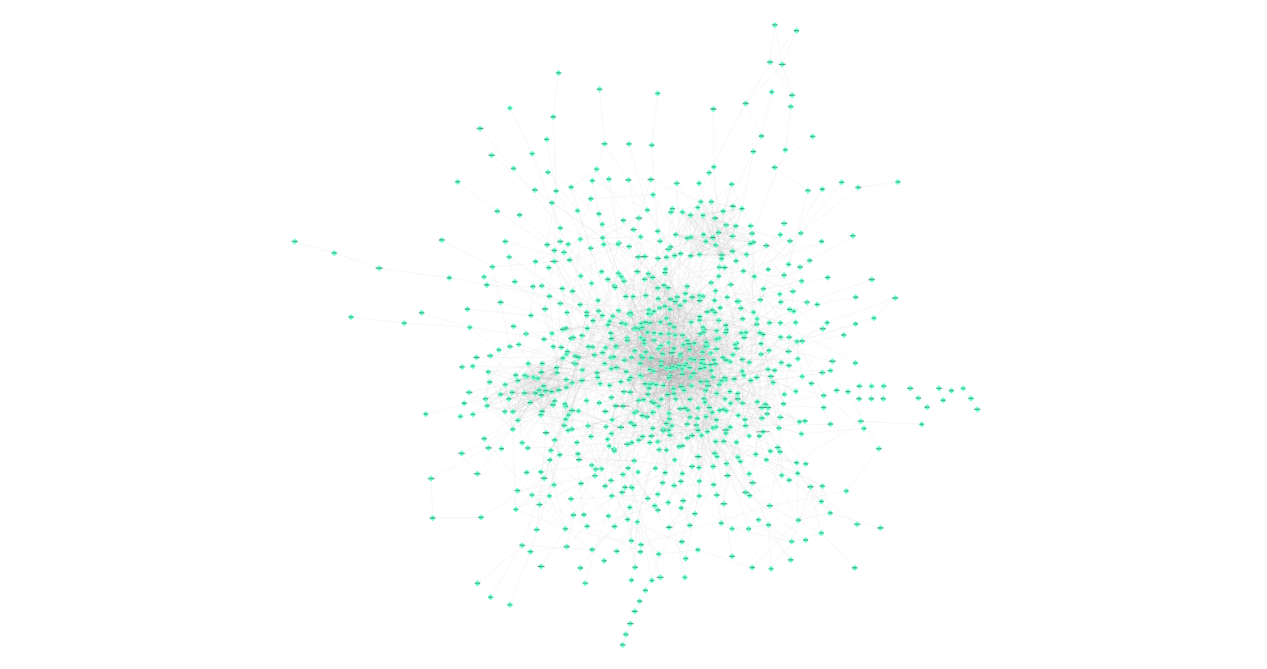


**Figure S4**. Protein-protein interaction (PPI) network of DEGs.
